# Specific members of the TOPLESS family are susceptibility genes for Fusarium wilt in tomato and Arabidopsis

**DOI:** 10.1111/pbi.14183

**Published:** 2023-10-11

**Authors:** Thomas R. Aalders, Mara de Sain, Fleur Gawehns, Nina Oudejans, Yoran D. Jak, Henk L. Dekker, Martijn Rep, Harrold A. van den Burg, Frank L.W. Takken

**Affiliations:** ^1^ Molecular Plant Pathology Swammerdam Institute for Life Sciences (SILS), University of Amsterdam Amsterdam the Netherlands; ^2^ Mass Spectrometry of Biomolecules Swammerdam Institute for Life Sciences (SILS), University of Amsterdam Amsterdam the Netherlands

**Keywords:** susceptibility gene, disease resistance, SIX8 effector, *Fusarium oxysporum*, virulence

## Abstract

Vascular wilt diseases caused by *Fusarium oxysporum* are a major threat to many agriculturally important crops. Genetic resistance is rare and inevitably overcome by the emergence of new races. To identify potentially durable and non‐race‐specific genetic resistance against Fusarium wilt diseases, we set out to identify effector targets in tomato that mediate susceptibility to the fungus. For this purpose, we used the SIX8 effector protein, an important and conserved virulence factor present in many pathogenic *F. oxysporum* isolates. Using protein pull‐downs and yeast two‐hybrid assays, SIX8 was found to interact specifically with two members of the tomato TOPLESS family: TPL1 and TPL2. Loss‐of‐function mutations in *TPL1* strongly reduced disease susceptibility to Fusarium wilt and a *tpl1;tpl2* double mutant exerted an even higher level of resistance. Similarly, Arabidopsis *tpl;tpr1* mutants became significantly less diseased upon *F. oxysporum* inoculation as compared to wildtype plants. We conclude that *TPLs* encode susceptibility genes whose mutation can confer resistance to *F. oxysporum*.

## Introduction

The fungal pathogen *Fusarium oxysporum* is a major threat to global food security (Chaloner *et al*., 2021; Edel‐Hermann and Lecomte, 2019; Gordon, 2017). The *F. oxysporum* species complex is able to infect over 100 different plant species, including economically important field crops, for example soybean, cotton, banana—vegetable crops, for example spinach, melon, tomato—and ornamentals, for example cyclamen, chrysanthemum and tulip (Edel‐Hermann and Lecomte, 2019). Control measures applied to infested fields are often impracticable or ineffective; the most effective method to control the pathogen is the use of resistant varieties. The development of resistant plant varieties by traditional breeding typically relies on introgression of naturally occurring, dominant resistance (*R*) genes, into cultivars. However, only for a limited number of crop species introgressable resistance sources are available in the wild or cultivated germplasms limiting their applicability. *R* genes mediate resistance by recognition of pathogen‐derived effector proteins (Bailey‐Serres *et al*., 2019; Kourelis and van der Hoorn, 2018). However, *R* gene‐mediated resistance is eventually broken by the emergence of new races that evade recognition, for instance, by mutations in the cognate effector protein or loss thereof (Dangl *et al*., 2013).

As an alternative, durable genetic resistance against plant pathogens can be established via inactivation of so‐called susceptibility (*S*) genes, which are plant genes that facilitate the infection process and support compatibility with the pathogen (van Schie and Takken, 2014; Zaidi *et al*., 2018). To overcome such resistance, mutations or gene loss on the pathogen side do not suffice; the pathogen is required to establish a new infection strategy independent of the host *S* gene product. *S* genes often have important functions for the plant. Hence, besides disease resistance their inactivation can result in pleiotropic effects such as reduced vigour, plant deformation or reduced fruit production (Garcia‐Ruiz, 2018; Garcia‐Ruiz *et al*., 2021; Pavan *et al*., 2010; van Schie and Takken, 2014). Notwithstanding these potential drawbacks, *S* genes hold great potential for application, as exemplified by *MILDEW RESISTANCE LOCUS O* (*MLO*). Loss‐of‐function mutant alleles of *MLO* confer broad‐spectrum powdery mildew (PM) resistance in barley, with little pleiotropic effects, for over 50 years (Freisleben and Lein, 1942; Kusch and Panstruga, 2017). *MLO* genes are present as small families in the genomes of all higher plant species. Their function in plant immunity is evolutionary conserved as specific *mlo* mutants also confer PM resistance in crops such as wheat, tomato, pea and grapevine (Acevedo‐Garcia *et al*., 2014). Another example of a ubiquitously used *S* gene is *eukaryotic initiation factor 4E* (*eIF4E*) (Sonenberg *et al*., 1978), which is essential for viral RNA replication. Inactivation of *eIF4E* leads to potyvirus resistance, and loss‐of‐function *eIF4E* mutans confer resistance in barley, rice, tomato, lettuce, pea, pepper and melon (Wang and Krishnaswamy, 2012). Inactivation of one of its isoforms, eIF4E or elF(iso)4E, is often sufficient for potyvirus resistance and evades pleiotropic effects (Charron *et al*., 2008). The functions of *S* genes are often conserved in plants as exemplified above and as shown for *SlDMR6‐1*. This tomato orthologue of *DOWNY MILDEW RESISTANCE 6*, which was originally identified in Arabidopsis, also confers broad‐spectrum disease resistance when inactivated in tomato (de Toledo Thomazella *et al*., 2021).

Although most S genes have been identified via forward genetics, an alternative approach to identify susceptibility factors is to screen for host targets of conserved pathogen effectors (Gawehns *et al*., 2013). Currently, no Fusarium *S* genes are known that can be applied in agri‐ or horticulture. To identify an S gene against *F. oxysporum*, we performed an effector‐host target screen using the tomato*—F. oxysporum* forma specialis (f. sp.) *lycopersici* (Fol) pathosystem (Takken and Rep, 2010). The Fol genome encodes a relatively small effectorome, including 14 SECRETED IN XYLEM (SIX) effectors (Houterman *et al*., 2009). We used the effector SIX8_Fol_ (hereafter referred to as SIX8) to identify interacting proteins because (i) *SIX8* is upregulated during infection (Sun *et al*., 2022; van der Does *et al*., 2016), (ii) *SIX8* is present in at least 14 different formae speciales (ff. spp.) capable of infecting various important crops such as tulip, banana, lettuce, melon and spinach (Table S1) and (iii) *SIX8* orthologs are absent in non‐pathogenic isolates such as Fo47 (Constantin *et al*., 2021; van Dam *et al*., 2016). SIX8 does not suppress general plant immunity triggered by bacterial and fungal elicitors flg22 or chitin, implying that SIX8 has a distinctive function during infection (Tintor *et al*., 2020). Here, we identified a host target of SIX8: TOPLESS—and show that specific members of this gene family act as susceptibility genes for *Fusarium* wilt disease in tomato and Arabidopsis.

## Results

### SIX8 interacts specifically with tomato TPL1 and TPL2

To identify host targets of the *F. oxysporum* SIX8 effector protein pull‐down experiments were performed using transiently transformed *Nicotiana benthamiana* leaves. As a negative control the sequence‐unrelated effector SIX6 was included (Gawehns *et al*., 2014). To prevent secretion, the endogenous signal peptides were deleted. Both effectors were C‐terminally fused to a HASBP‐tag (Hemagglutinin and Streptavidin‐binding protein) to aid purification. The co‐immunoprecipitated proteins were subjected to tryptic digestion and analysed by LC–MS/MS. Four peptides were uniquely identified in the SIX8 samples: two matched SIX8 and two peptides (LIEANPLFR and TLINQSLNWQHQLCK) corresponded to the C‐terminal regions of 12 out of the 13 TOPLESS‐related proteins present in the NbDE proteome database (Data S1) (Kourelis *et al*., 2019). TPR proteins are named after the founder TOPLESS (TPL) protein from *Arabidopsis thaliana*. Members of the TPL/TPR protein family act as negative co‐regulators of gene expression controlling a wide array of plant processes impacting (a)biotic stress, but also development and hormone responses (Causier *et al*., 2012; Ke *et al*., 2015; Long *et al*., 2002; Plant *et al*., 2021).

**Figure 1:**
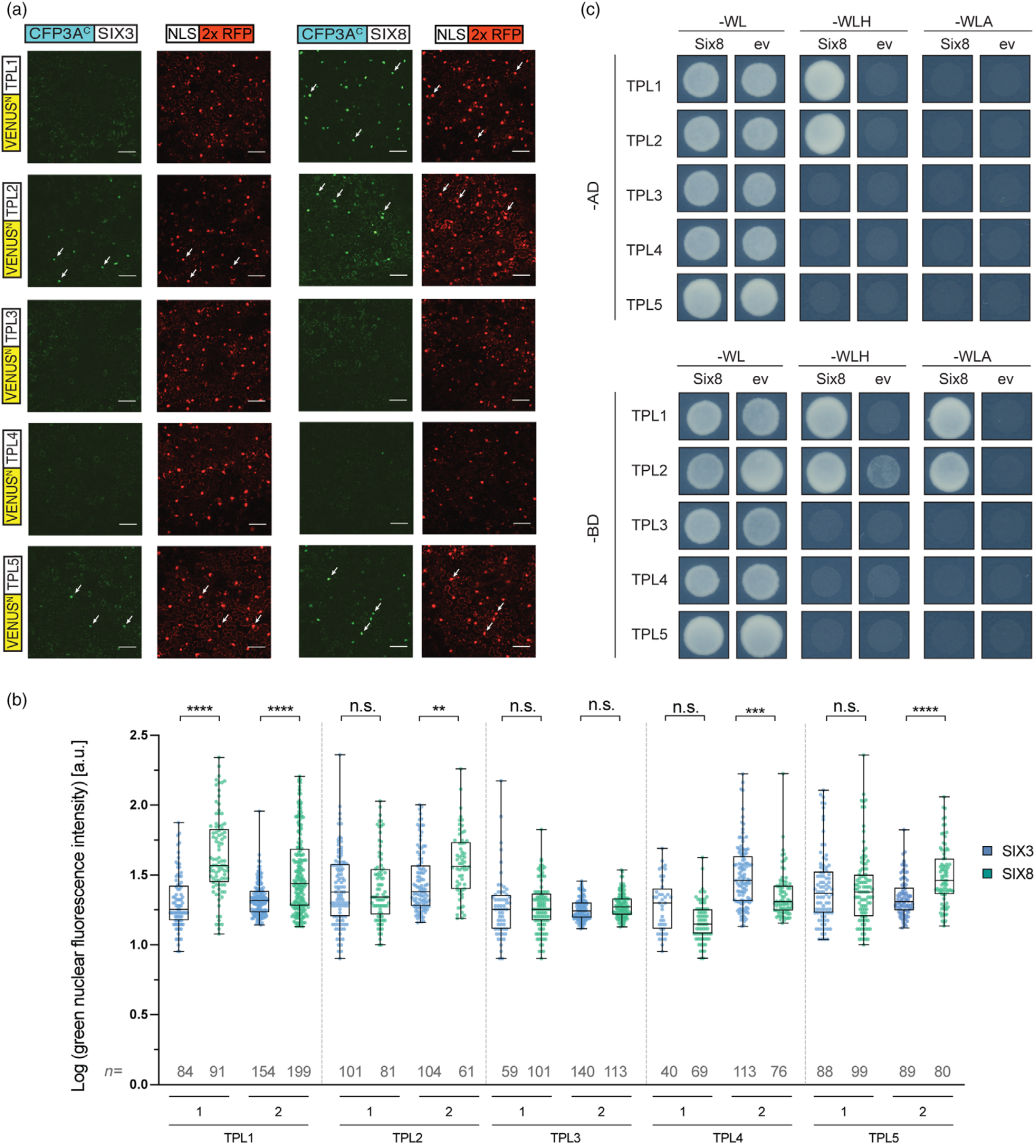
SIX8 interacts with TPL1 and TPL2. (a) BiFC assay in transiently transformed *N. benthamiana* leaves. Co‐agroinfiltration was performed with (i) *NLS‐2xRFP* fusion as a fluorescent nuclear marker and (ii) five *TPL* family members from tomato (*TPL*1 to *TPL*5) N‐terminally fused to *VENUS* ^*N*^ in combination with (iii) either *CFP3A* ^*C*^ *‐ΔspSIX8* or *CFP3A* ^*C*^ *‐ΔspSIX3* as a negative control. White arrows exemplify transformed nuclei (RFP) with a positive BiFC signal (GFP). (b) Quantification of the levels of green fluorescence in the nuclei following BiFC. The figure depicts two independent replicates. Significance tested by Kruskal–Wallis tests: **P ≤ 0.01, ***P ≤ 0.001, ****P ≤ 0.0001. (c) Yeast two‐hybrid assays of tomato TPL1 to TPL5 with either Fol SIX8 or an empty vector (ev) control. Bottom panel: same as (b) but bait (‐BD) and prey (‐AD) are switched.

To assess whether the tomato TPL homologues could be targets of SIX8, their potential interaction was analysed *in planta* using bi‐molecular fluorescence complementation (BiFC). The tomato genome encodes six TPL homologues: *TPL1* to *TPL6* (Hao *et al*., 2014). Interactions of SIX8 with TPL homologues 1 to 5 were analysed (not TPL6), as these five TPLs are (i) root expressed (Figure S1), (ii) nuclear localized and (iii) shown to interact with known TPL interactors such as Aux/IAA transcription factors (Hao *et al*., 2014). The TPLs were N‐terminally fused to the N‐terminal half of a yellow fluorescent protein (YFP) variant VENUS^N^. As negative control SIX3 (also known as Avr2) was used because (i) it has a similar molecular weight as SIX8, (ii) it exerts a nucleo‐cytoplasmic distribution, and (iii) Avr2 targets processes independent of TPLs (Blekemolen *et al.*, 2023; Cao *et al*., 2018; Ma *et al*., 2013). The effectors, without their signal peptide, were N‐terminally fused to the C‐terminal half of a cyan fluorescent protein (CFP): *CFP3A* ^*C*^ *‐ΔspSIX8* and *CFP3A* ^*C*^ *‐ΔspSIX3* (Ma *et al*., 2013). Upon interaction of the chimeric proteins a green‐fluorescent protein is reconstituted. The gene fusions were transiently co‐expressed in *N. benthamiana* leaves to assess a potential interaction of the encoded proteins. TPL protein accumulation and size was confirmed by immunoblotting (Figure S2, VENUS^N^‐TPLs ~150 kDa, *CFP3A* ^*C*^‐SIX8 ~25 kDa, *CFP3A* ^*C*^‐SIX3 ~28 kDa). To label transformed cells an *NLS‐2xRFP* construct was co‐expressed resulting in red fluorescent nuclei (Figure 1a). Bright green‐fluorescent nuclei, indicative of protein–protein interactions, were primarily observed when *SIX8* was co‐expressed with *TPL1*, *TPL2* or *TPL5* (Figure 1a). Of note, weak green fluorescence was observed in all samples. This is likely caused by protein crowding in the nucleus leading to self‐association of GFP protein halves (Tunc‐Ozdemir *et al*., 2016). Therefore, we quantified the intensity of the GFP signal in transformed cells as a proxy for interaction strength of the two proteins (Figure 1b). In both replicates the GFP intensity in *VENUS* ^*N*^‐*TPL1* expressing nuclei was found to be significantly higher in the *CFP3A* ^*C*^ *‐ΔspSIX8* samples than in *CFP3A* ^*C*^ *‐ΔspSIX3* controls, suggesting a SIX8‐TPL1 interaction. For *VENUS* ^*N*^‐*TPL3* and *VENUS* ^*N*^‐*TPL4* the green fluorescence was identical or lower than that of the negative control, implying that SIX8 does not interact with TPL3 or ‐4. For both *VENUS* ^*N*^‐*TPL2* and *VENUS* ^*N*^‐*TPL5* a signal higher than the negative control was only observed in one replicate, which identifies these as plausible targets of SIX8. Together, the BiFC experiments indicate that SIX8 localizes to the plant nucleus where it interacts with TPL1, TPL2 and TPL5, but not TPL3 and TPL4.

To further investigate the specificity and to test for a direct interaction between SIX8 and TPL1 to TPL5, GAL4‐based yeast two‐hybrid (Y2H) assays were performed. All genes were tested in both prey (pDEST22 or ‐AD) and bait (pDEST32 or ‐BD) vectors and interactions were scored by assessing yeast growth on selective plates (‐WLH or ‐WLA). SIX8 was found to specifically interact with TPL1 and TPL2 and not with the other TPLs. Irrespective of whether TPL1 and TPL2 were expressed as bait or prey, yeast growth was observed on selective ‐WLH plates in the presence of SIX8 (Figure 1c). When provided as bait, both TPLs also complemented growth on the more stringent ‐WLA selection, suggesting a strong and specific interaction between SIX8 and TPL1 and ‐2. Of note, BD‐TPL2 in combination with the ev (pDEST22) showed some growth on ‐WLH selection, indicating a weak auto‐activity of TPL2. No growth was observed for other TPLs or the empty vector (ev) controls. Protein accumulation and size were confirmed by immunoblotting (Figure S3) (AD‐TPLs ~140 kDa). In conclusion, together the BiFC and Y2H data imply a specific, direct and nuclear‐localized interaction between SIX8 with TPL1 and TPL2.

### TOPLESS loss‐of‐function mutants confer resistance to *Fusarium oxysporum* f. sp. *lycopersici*, but do not affect susceptibility to *Verticillium dahliae* or *Pseudomonas syringae*

**Figure 2:**
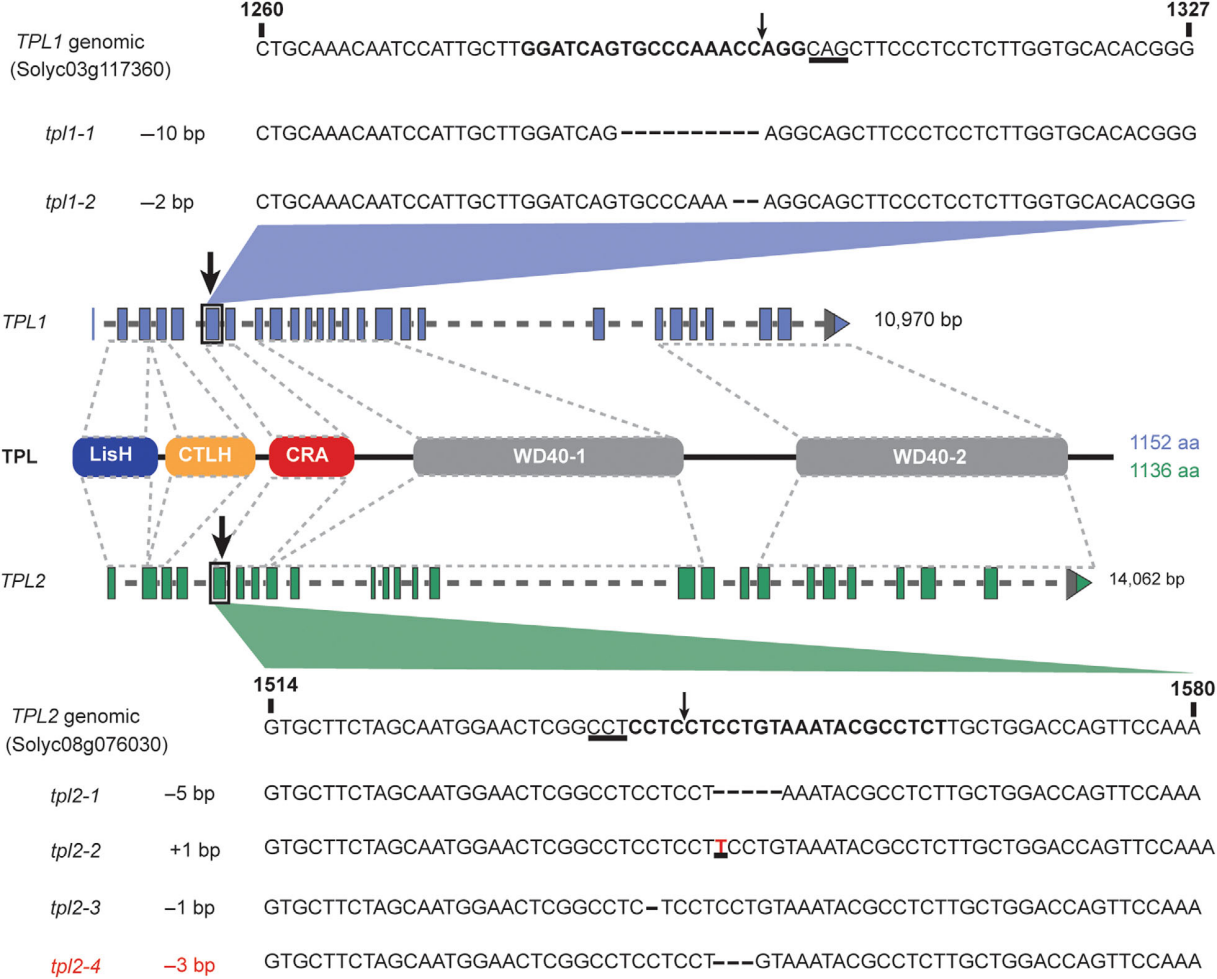
Mutant alleles of tomato *TPL1* and *TPL2* generated by CRISPR‐Cas9‐mediated gene editing. Two gene‐edited alleles of *TPL1* were generated by targeting the 6th exon with a gRNA. The TPL protein domains are indicated in the depicted protein model (not to scale): LisH (Lissencephaly type‐1‐like homology motif), CTLH (C‐terminal LisH domain) and two WD40 repeat regions. Four gene‐edited alleles of *TPL2* were generated by targeting the 5th exon. The functional gRNA sequences are highlighted in bold. The PAM sequence is underlined, the predicted cleavage site is indicated with a black arrow. Gene edits are indicated by a dash for each deleted nucleotide or a red letter for an inserted nucleotide.

To examine the role of *TPL1* and *TPL2* in susceptibility of tomato towards Fol, targeted knockouts were generated in the susceptible variety Motelle using CRISPR‐Cas9‐mediated gene editing (Cermak *et al*., 2017; Figure 2). Stable transgenic lines were generated co‐expressing the *CAS9* gene and four guideRNAs (gRNAs); two gRNAs per *TPL* gene. To ensure functional disruption of the gene product, the region encoding the C‐terminal CT11 RanBPM (CRA) domain was targeted. Small indels were observed in the targeted TPLs showing that one of the two gRNAs per *TPL* gene was working. Out of 67 lines analysed, one *tpl1*, two *tpl2's* and three *tpl1;tpl2* double mutants were identified (Table S2). In our greenhouse growth and development of the *tpl* single and double mutants was like that of the progenitor line. No apparent phenotypic differences were observed between the single *tpl1* and *tpl2* mutants and wildtype plants. However the flowers of the *tpl1;tpl2* double mutant were slightly larger than those of the progenitor (Figure S4). Furthermore, although fruit number and yield of the Motelle variety was similar as that of the *tpl1;tpl2* mutants the latter produced less seeds. Wildtype plants produced ~27.15 seeds per fruit, while both double mutants *tpl1‐2;tpl2‐2* and *tpl1‐2;tpl2‐3* produced, respectively, ~4.74 and 3.06 seeds per fruit (Figures S5 and S6).

**Figure 3:**
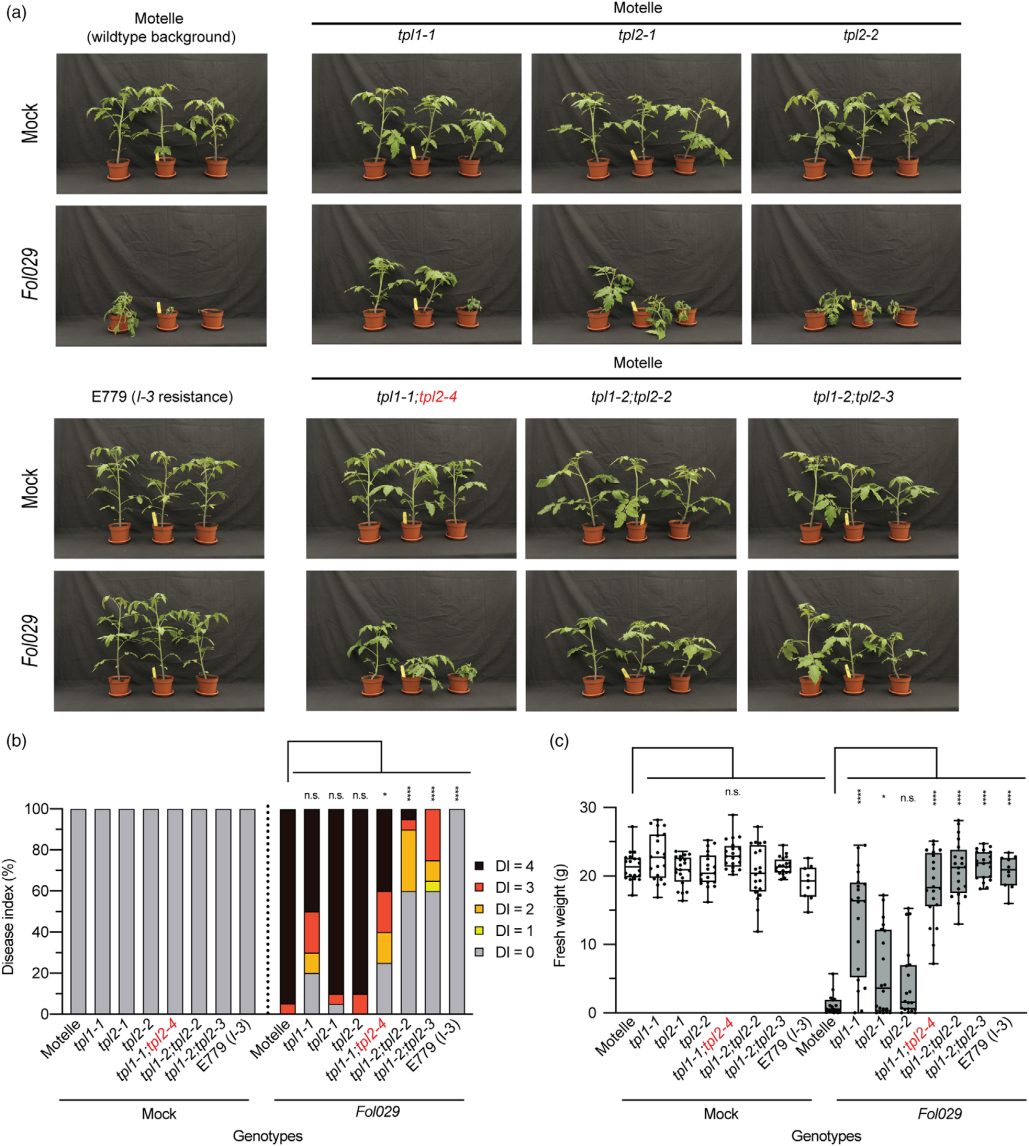
Gene‐edited tomato *tpl* mutants are less susceptible to *Fusarium oxysporum*. (a) Photographs of tomato plants inoculated with Fol029 or sterile water (Mock), 21 days post‐inoculation. (b) Disease index scores based on plant size and vascular browning. (c) Tukey boxplots (min–max) of plant fresh weight above the cotyledon, individual data points are indicated by black dots. Significance tested by ANOVA followed by *t*‐tests with Dunnett's multiple testing correction: n.s., non‐significant, *P ≤ 0.05, ****P ≤ 0.0001. The assay was repeated four times with similar results.

To assess disease susceptibility, the *TOPLESS* mutants were inoculated with *F. oxysporum* race 3 isolate Fol029. The susceptible progenitor (Motelle) and a resistant variety (E779) carrying the *I‐3* resistance gene were included as controls (Rep *et al*., 2005). Seedlings were inoculated with Fol029 or mock‐treated, and disease symptoms and plant weight were scored 3 weeks post‐inoculation. As expected, wildtype Motelle plants challenged with Fol029 showed severe wilt disease symptoms, including stunting, yellowing of the leaves and wilting, while the resistant E779 variety remained healthy (Figure 3a). Disease index (DI) and plant weight of the *tpl2‐1* and *tpl2‐2* single mutants inoculated with Fol029 were indistinguishable from those of the susceptible wildtype controls (Figure 3b,c). The biomass of the *tpl1*‐*1* single knockout, however, was significantly increased implying a reduced susceptibility, although the DI was like that of the susceptible control. The *tpl1‐1;tpl2‐4* double mutant showed a DI and fresh weight similar to the *tpl1‐1* single mutant, indicating that the *tpl2‐4* mutant allele (−3 bp; TPL2ΔP215) is not a loss‐of‐function mutation. Notably, compared with the susceptible controls the Fol029‐inoculated *tpl1‐2;tpl2‐2* and *tpl1‐2;tpl2‐3* double mutants displayed both increased weight and a strong reduction in DI (Figure 3b,c). Resistance of these *tpl1/tpl2* double mutants was indistinguishable to that of the E779 resistant control showing that these *tpl's* are genuine *S* genes. Taken together, a loss‐of‐function mutation in *TPL1* suffices to reduce susceptibility to Fol029, but near complete resistance was obtained when *tpl1* and *tpl2* mutants were combined.

To assess whether susceptibility to another vascular fungal pathogen was altered in the *tpl* lines, disease assays were performed using *Verticillium dahliae*. Ten‐day‐old seedlings were root‐dip inoculated with either mock or spores of *V. dahliae* (Dvd‐S26). Disease progression was scored at 14 days post‐inoculation by measuring the canopy area. In general, Dvd‐S26 inoculated plants had a smaller canopy than the mock controls (Figure S7). Although among the three repetitions differences in canopy areas were occasionally observed for some *tpl* lines, for example, for *tpl2‐1*, these differences were not consistent over the replicates. Also because a reduction of canopy area was not observed in the other *tpl2* (*tpl2‐2)* mutant, we conclude that TPL1 and/or TPL2 do not affect susceptibility to *V. dahliae*.

Earlier, it was reported that *A. thaliana*, *tpl;tpr1* and *tpl;tpr1;tpr4* triple mutants are more susceptible towards infection with *P. syringae* strain DC3000 (Zhu *et al*., 2010). To test whether loss‐of‐function mutations in tomato *TPL1* and *TPL2* affect susceptibility to *P. syringae* DC3000, disease assays were performed. *In vitro* grown tomato seedlings were submerged in a bacterial inoculum (OD_600_ = 0.005) and cultivated for 4 days (Hassan *et al*., 2020). Host colonization was quantified by determining bacterial titres in the infected cotyledons (Figure S8). No significant differences between the wildtype cultivar and the *tpl* mutants were found, showing that *tpl1* and *tpl2* are not involved in susceptibility to *P. syringae* DC3000 in tomato.

### *tpl‐*mediated resistance to *F. oxysporum* is root‐based and graft‐transmissible

**Figure 4:**
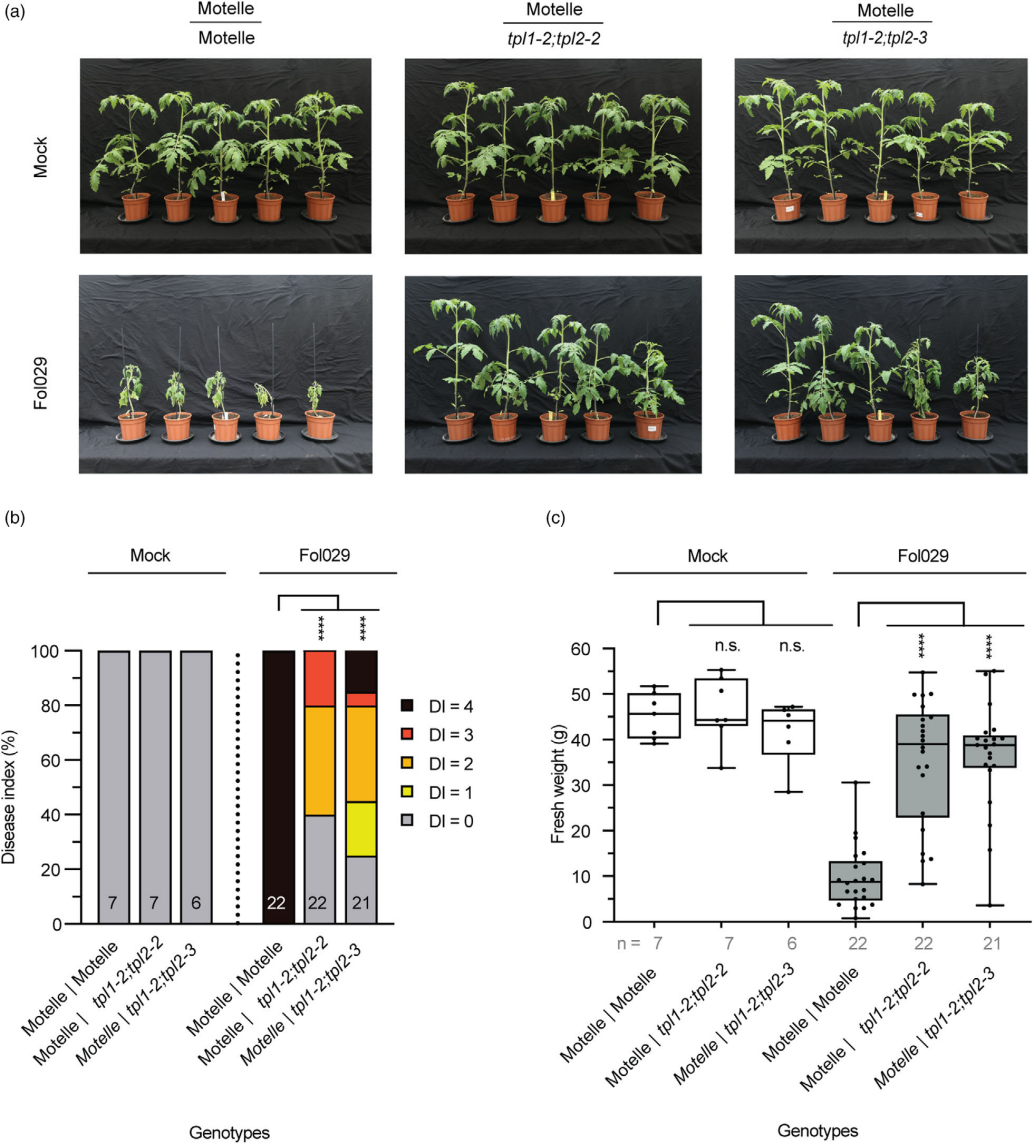
Grafting susceptible tomato scions on *tpl1;tpl2* rootstocks confers resistance to *Fusarium oxysporum*. Wildtype scions (cv. Motelle) were placed on rootstocks of Motelle, *tpl1‐2;tpl2‐2* or the *tpl1‐2;tpl2‐3* double mutant. Plants were either mock‐inoculated (sterile water) or inoculated with Fol029, using the root‐dip method. (a) Representative pictures of plants 3 weeks post‐inoculation. (b) Bar graph showing disease index of the (grafted) genotypes (scion|rootstock). Significant differences were determined using a Kruskal–Wallis rank test by comparison to wildtype (cv. Motelle). (c) Tukey boxplots (min–max) of plant fresh weight above the cotyledons, all data points are indicated by black dots. Significance tested by ANOVA followed by *t*‐tests with Dunnett's multiple testing correction: Significance is indicated: n.s., non‐significant, ****P ≤ 0.0001. This assay was replicated three times, with similar outcomes.

Greenhouse‐grown tomatoes are commonly grafted on resistant rootstocks to protect the elite scion against infection with *F. oxysporum* or other pathogens (Thies, 2021). To determine whether *tpl1;tpl2*‐ rootstocks can protect a susceptible scion, wildtype (cv. Motelle) scions were grafted onto *tpl1‐2;tpl2‐2* and *tpl1‐2;tpl2‐3* rootstocks (scion|rootstock) (Figure 4a). Subsequently, the chimeric plants were either mock‐ or Fol029*‐*inoculated, and fresh weight and DI were scored. The fresh weight of mock‐inoculated wildtype|wildtype grafts was identical of that of mock‐inoculated wildtype|*tpl1;tpl2* plants. As expected Fol029‐inoculated wildtype|wildtype grafts showed severe disease symptoms, resulting in a disease index score of 4 (Figure 4b). In contrast, Fol029‐inoculated plants carrying a *tpl1;tpl2* mutant rootstock were more vigorous and exhibited fewer *F. oxysporum*‐related disease symptoms than those on wildtype rootstocks. Actually, fresh weight of the former was similar to that of mock‐treated chimeras (Figure 4c) even though brown vessels were frequently observed. Brown vessels suggest the presence of the pathogen in the vessels. The presence of Fol029 in the vasculature was assessed by placing stem pieces on Potato Dextrose Agar plates. Whereas all stem pieces taken at the 2nd true leaf height of wildtype|wildtype plants showed fungal outgrowth, wildtype|*tpl1;tpl2* grafted chimeras showed significant less colonization (Figure S9). These data show that *tpl1;tpl2* confers systemic root‐based resistance to *F. oxysporum* protecting a susceptible scion against disease development. The resistance of the grafted chimera is identical to that of the *tpl1;tpl2* double mutants.

### TOPLESS loss‐of‐function mutants confer resistance against *F. oxysporum* isolate Fo5176 in *Arabidopsis thaliana*

**Figure 5:**
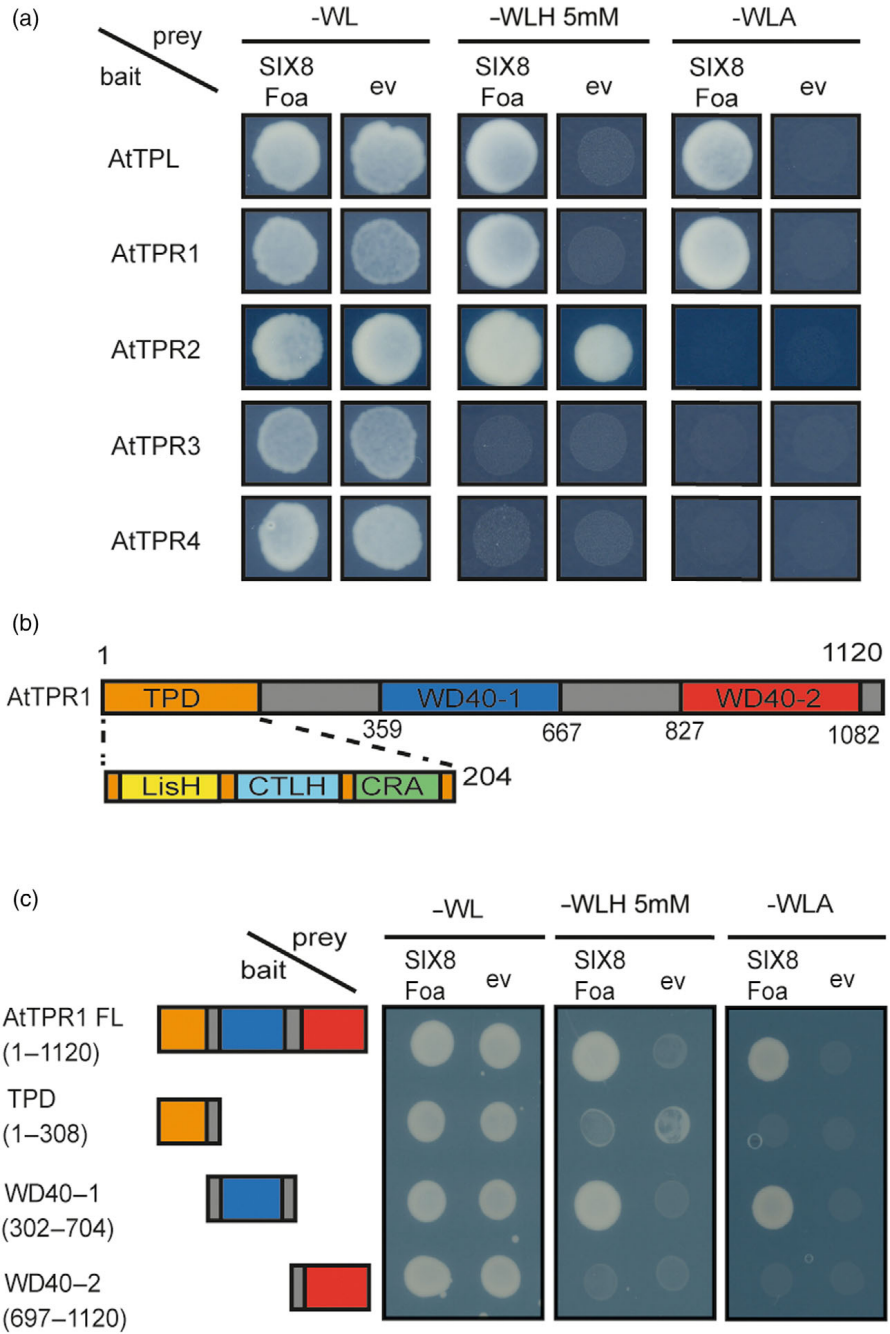
SIX8 from Fo5176 interacts with the WD40‐1 repeat of Arabidopsis TPR1. (a) Yeast two‐hybrid assays of TPL and TPR1‐T4 from *A. thaliana* with Foa SIX8 or an empty vector (ev) control. (b) Schematic drawing of the domain structure of TPR1, with an N‐terminal TOPLESS (TPD) domain, consisting of Lissencephaly Homologue (LisH) domain, a C‐terminal to LisH (CTLH) domain and CT11‐RanBPM (CRA) domain. Towards the C‐terminus of the protein the two WD40 repeat domains (WD40‐1 and WD40‐2) are depicted. Numbers indicate amino acid position. (c) Yeast two‐hybrid assays of individual TPR1 protein domain fragments with Foa SIX8 or an empty vector (ev) control. Yeast containing bait and prey plasmid grows on selective medium lacking ‐WL. Growth on ‐H or ‐A is indicative for an interaction between bait and prey proteins.

Considering the omnipresence of *TPL*s in land plants, and the conservation of *SIX8* in the *F. oxysporum* species complex (Table S1), we hypothesized that *TPL* homologues may also act as *F*. *oxysporum* susceptibility genes in another plant species. *F. oxysporum* strain Fo5176 (Foa) causes wilt disease in *A. thaliana*, and a *SIX8* knockout is compromised in virulence (Ayukawa *et al*., 2021). To test whether the five Arabidopsis TPLs: *AtTPL* and the four *TOPLESS‐related (TPR)* gene products, interact with SIX8_Foa_ Y2H assays were performed. Correct size and accumulation of the chimeric proteins was confirmed by immunoblotting (Figure S10) (Tagged‐TPL/TPRs ~130–140 kDa). Yeast growth on ‐WLH plates was observed when TPL, TPR1 and TPR2 were combined with SIX8_Foa_ (Figure 5a). Some growth was observed for the empty vector control for TPR2 indicating weak auto‐activity of the *pDEST32‐TPR2* construct. No growth was observed for TPR2 on more stringent ‐WLA selection, while TPL and TPR1 retained their ability to grow suggesting a specific interaction of the latter proteins with SIX8_Foa_. To determine the interaction site of SIX8_Foa_ with TPR1, the protein was separated into its three functional domains: a N‐terminal TOPLESS (TPD) domain and the two WD40‐repeat regions (WD40‐1 and WD40‐2) (Figure 5b). Full‐length TPR1 and the three fragments, each compromising one of the three domains, were tested in a Y2H assay (Figure 5c). The correct size and accumulation of the chimeric proteins was confirmed by immunoblotting (Figure S10). Yeast growth was observed when SIX8_Foa_ was combined with full‐length TPR1 and with the first WD40 repeat domain (WD40‐1) on both ‐WLH and ‐WLA plates suggesting a strong interaction (Figure 5c). These data show that SIX8_Foa_ interacts specifically with the first WD40 domain of TPR1.

**Figure 6:**
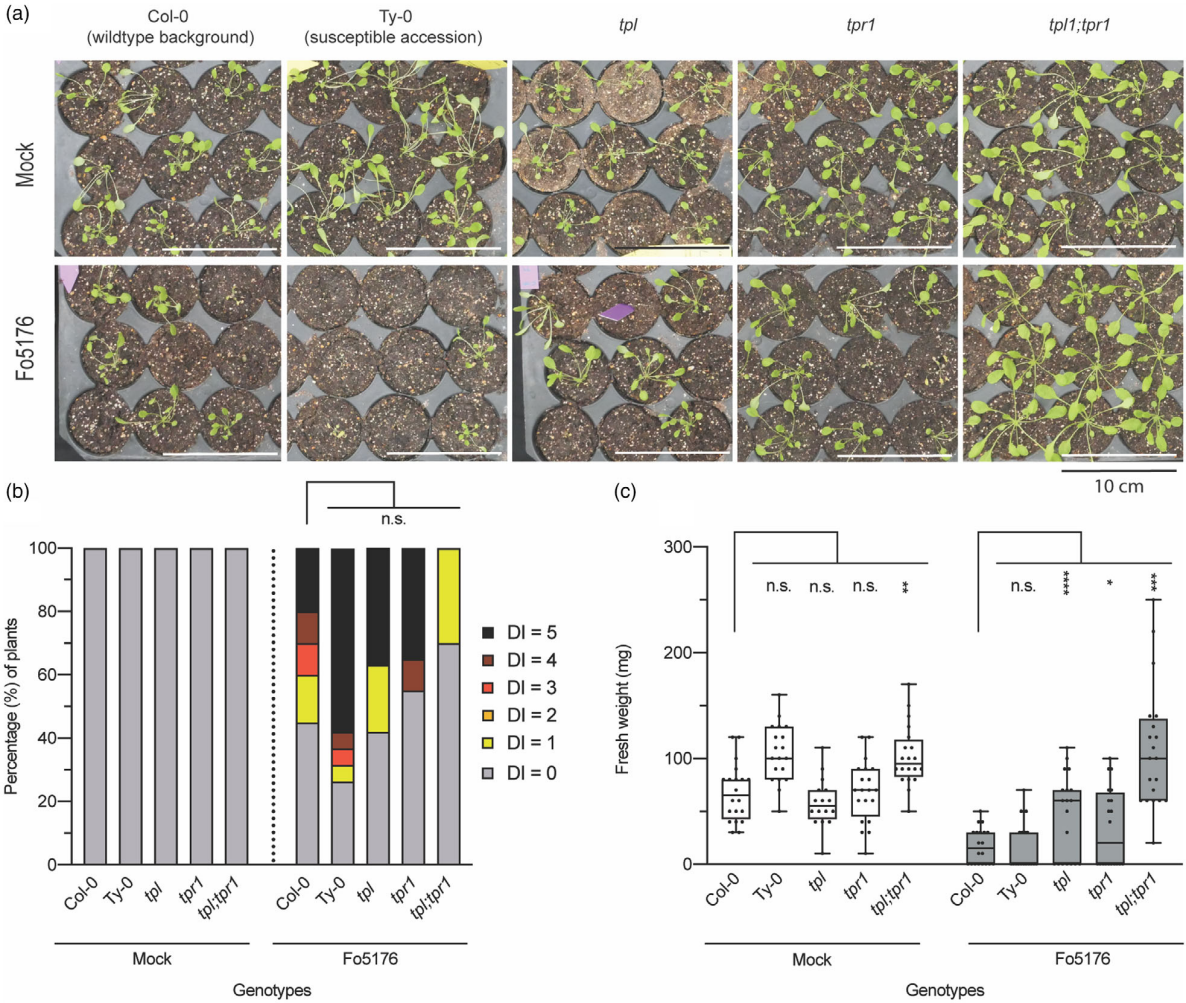
Arabidopsis *tpl* mutants are less susceptible to *Fusarium oxysporum* Fo5176. (a) Representative photographs of Arabidopsis seedlings inoculated with Fo5176 or water (Mock), 14 days post‐inoculation. The elevated ambient temperature required for consistent infection resulted in etiolated plants. (b) Disease index scores based on plant size and leaf/vein chlorosis. (c) Tukey boxplots (min–max) of plant fresh weight, individual data points are indicated by black dots. Significance tested by ANOVA followed by *t*‐tests with Dunnett's multiple testing correction: n.s., non‐significant, *P ≤ 0.05, **P ≤ 0.01, ***P ≤ 0.001, ****P ≤ 0.0001. The assay was repeated four times with similar outcomes. DI was scored in all replicates while plant weight was recorded in two of the four replicates.

To test whether the SIX8‐interacting TPL/TPRs also contribute to disease susceptibility in Arabidopsis Col‐0, single *tpl* and *tpr1* mutants and the *tpl;tpr1* double were inoculated with *F. oxysporum* isolate Fo5176. The relative resistant progenitor Col‐0 and the hypersusceptible accession Ty‐0 were included as controls (Diener and Ausubel, 2005). Following Fo5176 inoculation Ty‐0 plants developed strong disease symptoms such as vascular chlorosis, growth retardation and tissue collapse (Figure 6a). Around 60% of the inoculated Ty‐0 plants had a maximum DI score of 5 (Figure 6b). Col‐0 plants showed less disease symptoms; only 20% had a DI of 5 and over half showed a DI ≧ 1. The DI of the Fo5176 inoculated *tpl* single mutant was like that of the Col‐0 control, however, plant weight was significantly higher indicating reduced susceptibility (Figure 6c). Also, the DI of the inoculated *tpr1* single mutant was indistinguishable from Col‐0, while its biomass was significantly increased as compared to Col‐0. The DI of the inoculated *tpl;tpr1* double mutant, however, was significantly decreased; 70% of the plants showed no visible disease symptoms while the others had a score of 1. The fresh weight of the inoculated *tpl;tpr1* mutants was indistinguishable to that of the mock controls indicating an additive effect of the *TOPLESS* alleles on resistance. Interestingly, mock‐treated *tpl;tpr1* plants showed an increase in biomass as compared to Col‐0, indicating increased growth at 28 °C. This growth increase remained upon inoculation with Fo5176, resulting in an even larger increase in weight. Together these data show that *tpl* and *tpr1* reduce susceptibility towards Fo5176. The *tpl* allele contributes most strongly to resistance to Fo5176, but a combination of *tpl;tpr1* alleles confers an even higher reduction in susceptibility.

## Discussion

We report identification of an *S* gene against Fusarium wilt: the transcriptional co‐repressor *TOPLESS* and show that null mutants confer resistance in two different plant species. A tomato *tpl1* null mutation confers a modest level of disease protection, reflected by increased plant weight upon Fol infection. Whereas *tpl2* alone did not affect susceptibility to Fusarium, near full resistance was observed when combined with *tpl1*. Likewise, Arabidopsis single *tpl* and *tpr1* null mutants exerted mildly reduced disease susceptibility to Fo5176 while strongly reduced DI and increased plant weight was observed for the *tpl;tpr1* double mutant. Grafting experiments revealed that *tpl1;tpl2‐*mediated resistance is root‐based, which corresponds with the infection process of the soil‐born root invading fungus (Figure 4).

Plant growth and development of the single and double mutants of the SIX8‐interacting TPLs—in both Arabidopsis and tomato—was like that of wildtype plants. The absence of an apparent major phenotype in plants in which two important transcriptional co‐regulators are compromised, was surprising and might be explained by functional redundancy conferred by non‐SIX8‐interacting TPLs. This would be reminiscent to the *ElF4a* and *MLO S* genes, where the pathogen relies on specific family members that are redundant for the host—but essential for pathogenesis. A loss‐of‐function mutation in the required allele compromises susceptibility without (severely) affecting host performance. *TOPLESS* genes encode transcriptional co‐repressors with important functions. These co‐repressors control gene expression involved in various cellular processes including plant development, hormonal pathways and (a)biotic stress responses (Causier *et al*., 2012; Liu and Karmarkar, 2008; Szemenyei *et al*., 2008; Zhu *et al*., 2010). Transcriptional repression is essential to orchestrate tissue specific, and developmentally regulated gene expression patterns. To repress gene activity, TOPLESS proteins interact with other proteins to assemble repression complexes in the nucleus. Known interactors include mediator proteins (MED21, MED10), hundreds of different transcription factors (TFs) and histone modifiers such as histone deacetylases (HDA19) (Causier *et al*., 2012; Krogan *et al*., 2012; Leydon *et al*., 2021). Besides interacting with different partners, *TPL*s also differ among each other in their expression levels and ‐patterns (Figure S1). Although not all *TPL*s are expressed in all plant organs, typically multiple members are expressed in a given tissue. In tomato, *TPL1* is highly expressed in roots, leaves, flowers and young fruits (Figure S1). The lack of an apparent phenotype in the *tpl1* knockout—and the modest phenotype of the *tpl1/tpl2* mutant—implies that the other *TPLs* expressed in these tissues take over its function.

Functional inactivation of *S* genes frequently results in pleiotropic phenotypes (van Schie and Takken, 2014). Arabidopsis *tpl;tpr1* mutants were reported to be hypersusceptible to *Pseudomonas syringae* DC3000 and to avirulent isolates carrying *AvrRps4*, but not *AvrRpt2* (Zhu *et al*., 2010). This contrasts our findings in tomato, susceptibility to this bacterium was unaffected in both the single and the double *tpl1/tpl2* mutants. Likewise, susceptibility to *V. dahliae* was unaltered in the mutants implying that *tpl‐*mediated resistance in tomato might be Fusarium wilt specific. A mild developmental defect on seed production and flower size was observed, but only when multiple *TPL* genes were mutated (Figures S2–S4). Scions grafted on *tpl1;tpl2* rootstocks developed normal and did not display this phenotype, showing that resistance can be uncoupled from this potential trade‐off. For breeding purposes, a single *tpl1* mutant might suffice, as it confers a modest level of resistance to Fusarium wilt without any apparent phenotype. When grown at elevated temperatures (30/35 °C) silencing of *TPL1* in tomato was reported to increase fruit production by inducing facultative parthenocarpy (He *et al*., 2021). To assess the usefulness of the Fusarium *S* gene in agri‐ or horticulture, a detailed investigation of the responses of the single and double TOPLESS mutants exposed to various (a)biotic stresses is recommended.

The mechanism by which SIX8 manipulates TPLs to benefit the fungus is unknown. For the virulence function, we deem it unlikely that SIX8 (fully) compromises TPL activity, as it would phenocopy the *tpl1;tpl2* knockout resulting in increased resistance. Since a *tpl2* mutant is still fully susceptible and manipulation of tomato TPL1 by SIX8 suffices to cause disease, the effect must be dominant, also as it would otherwise be masked by other TPLs. Therefore, we hypothesize that SIX8 specifically enhances TPL‐mediated repression of specific target gene(s) that are disadvantageous for fungal proliferation. To identify such genes flower dip transformation of Arabidopsis was done with a CaMV*35S* promotor‐driven *SIX8*_*Fol*_ construct. This resulted in low numbers of transformants, which showed dwarfism and developmental defects. The autoimmune phenotype suggests that the effector might be recognized in Col‐0 preventing functional analyses of its virulence function and identification of the target genes of the SIX8‐TPL protein complex. Interestingly, besides *F. oxysporum*, various other phytopathogenic fungi, bacteria and oomycetes carry effectors that target TPLs, highlighting their involvement in susceptibility. MLP124017 from the rust fungus *Melampsora larici‐populina* interacts with TOPLESS‐related 4 (TPR4) from poplar (Petre *et al*., 2015). HaRXL21, from the oomycete *Hyaloperonospora arabidopsidis* interacts with TPL1 and TPR1 from Arabidopsis and affects expression of TPR1‐repressed genes (Harvey *et al*., 2020). Rhizogenic *Agrobacterium* contains RolB, an effector that targets TPL1, ‐2 and ‐4 in tomato, that plays a major role in the hairy root development. Interestingly, RolB contains two LxLxL Ethylene‐responsive element binding factor‐associated amphiphilic repression (EAR) motifs, that are required for binding the TPLs and for its the virulence function. *Ustilago maydis* contains multiple TPL‐binding effectors, Naked1 (Nkd1) binds TPL1, TPL3 and TPL4 from *Zea mays*, and *Nkd1*‐expression in Arabidopsis de‐repressed auxin‐ and jasmonate signalling (Navarrete *et al*., 2022). Moreover, *U. maydis* contains a cluster of six additional TOPLESS interacting proteins (Tips) that induce genes involved in the auxin pathway (Bindics *et al*., 2022). Tip1 to ‐4 bind the TPD region of different maize TPLs. Binding is dependent on the LxLxL EAR motif that is present in many TPR‐interacting transcription factors (TFs). Finally, Jsi1 from *U. maydis* interacts, through an DLNxxP‐type EAR motif with the C‐terminal WD40 domain of ZmTPL1 (Darino *et al*., 2020). This motif is important for TPL‐binding proteins, such as VRN5, to bind to WD40 domains (Collins *et al*., 2019). Interestingly, we did not detect any known TPL‐binding motif, including an EAR motif in SIX8, while the effector binds to the first WD40 domain of TPR1. The peptide motif or protein interaction surface involved in this binding remains to be elucidated. Moreover, heterologous *Jsi1* expression in Arabidopsis and *Z. mays* triggers the transcriptional induction of the ethylene response factor (ERF) branch of the jasmonate/ethylene signalling pathway. In tomato jasmonate appears not to be involved in Fol susceptibility, whereas ethylene has an important contribution identifying this signalling pathway as a potential target for SIX8 (Di *et al*., 2017b). The role of these hormones in susceptibility to *F. oxysporum* differs between plant species (Di *et al*., 2016).

*TOPLESS* orthologs are conserved among land plants, including *Picocystis spp*, *Chlorophyta*‐ and *Streptophyta* species that diverged over 1 billion years ago (Plant *et al*., 2021). It is tempting to speculate that *tpl*‐mediated resistance is translatable to other crops. Many of the genomes of *F. oxysporum* isolates that infect crops such as cabbage, lettuce, tulip, melon, spinach and banana carry *SIX8* genes (Table S1). A knockout of *SIX8* in the banana infecting *F. oxysporum* f. sp. *cubense* tropical race 4 (TR4) (also known as *F. odoratissimum*) strongly compromises fungal virulence (Dita *et al*., 2018; García‐Bastidas *et al*., 2020). If the mode of action of TR4 SIX8 resembles that of Fol SIX8, a knockout of banana SIX8‐interacting *TPL*s might also confer protection against this disease.

## Experimental procedures

### Arabidopsis plant materials and growth conditions

Loss‐of‐function Arabidopsis mutants for *TPL* (*tpl‐2;* SALK_097230; referred to as *tpl*) were obtained from the NASC collection. The *TPR1* (*tpr1‐1* [*mos10*]; referred to as *tpr1*) mutant was obtained from Zhu *et al*. (2010). The *tpl;tpr1* double mutant was generated by crossing *tpl* and *tpr1* single mutants and selecting homozygotes in the F2.

### Plasmid construction

The *TPL*1‐5 genes and *SIX6 and SIX8* genes, without encoding signal peptides, were PCR‐amplified from cDNA of Fol007‐infected tomato roots (cv. GCR161) (De La Fuente *et al*., 2005) using high‐fidelity Phusion (ThermoFisher) and sequence verified by Sanger sequencing (primers used are listed in Table S4). The gene fragments encoding SIX6_20–225_ and SIX8_18–141_ are referred to as *ΔspSIX6* and *ΔspSIX8*, respectively. The PCR fragments carrying *ΔspSIX8‐HASBP* and *ΔspSIX6‐HASBP* were ligated into the *Xba*I and *Bam*HI sites of pSLDB3104 (Tameling *et al*., 2010; Table S3). For SIX3 the entry vector pENTR207::SIX3 was used (Houterman *et al*., 2009). The *ΔspSIX8* gene fragment was equipped with *attB1* and *attB2* gateway‐recombineering sites by PCR using primers FP872 and FP873. Thereafter, *ΔspSIX8* was recombined into pDONR207using BP clonase II (ThermoFisher), resulting in pENTR207::*ΔspSIX8*.

*TPL*, *TPR1* and *TPR3* were amplified from plasmids kindly provided by Causier *et al*. (2012) (Table S3). *TPR2*, *TPR4* and *SIX8* were PCR amplified from cDNA isolated from Fo5176 infected Arabidopsis (Col‐0). *TPR1* fragments for Y2H analyses of were generated by PCR. All gene (fragments) were equipped with *attB1* and *attB2* sites by PCR using primers FP872 and FP873 and subsequently recombined into pDONR207 and/or pDONR221.

To fuse *ΔspSIX8* and *ΔspSIX3* to the C‐terminal half of CFP3A^C^ and the *TPL*s to the N‐terminus of Venus^N^, a Gateway‐compatible vector set was used (Gehl *et al*., 2009). BiFC destination vectors were obtained by performing LR clonase II reactions between pENTR207::*TPL1‐5* and pDEST‐VYNE(R)^GW^ and between pENTR207::*ΔspSIX8*, pENTR207::*ΔspSIX3* and pDEST‐SCYCE(R)^GW^. The pGWB454::*NLS‐2xRFP* (*NLS‐2xRFP*) construct was described previously (Cao *et al*., 2018).

The GAL4 yeast two‐hybrid vectors were generated by transferring *TPLs* sequences and *ΔspSIX8* sequence from their respective entry vectors to pDEST22 and pDEST32 or pDEST32_amp using LR clonase II (ThermoFisher). pDEST32_amp was generated by replacing the gentamicin resistance cassette for the ampicillin resistance cassette of pDEST22 following *Lgu*I digestion resulting in pDEST32_amp, to facilitate bacterial selection (Table S3).

### Transient expression and protein pull‐down of SIX8‐HASBP from *N. benthamiana* leaves

*Nicotiana benthamiana* plants were cultivated in a greenhouse (21 °C, 60% RH, 16/8 h day/night). Transient transformation was performed using *A. tumefaciens* GV3101 resuspended at an OD_600_ of 1.0 (Ma *et al*., 2012). *A. tumefaciens‐*infiltrated leaves were sampled at 24–48 hpi and frozen at −80 °C. 6 g of the frozen leaves was ground in liquid N_2_ using a mortar and pestle and thawn in 12 mL extraction buffer (25 mm Tris–HCl pH 8.0, 150 mm NaCl, 1 mm EDTA, 5 mm DTT, 1× Complete protease inhibitor cocktail (Roche), 2% w/v PVPP, and 0.1% v/v NP‐40). Cell debris was removed by pelleting at 17 000 *g* (30 min, 4 °C). The supernatant was filtered over Miracloth and 400 μL streptavidin (SA‐) Sepharose slurry was added to allow protein binding for 1 h at 4 °C on a rotating wheel. The beads were washed 10× in 500 μL washing buffer IPP150 (10 mm Tris–HCl pH 8.0, 150 mm NaCl, and 0.1% v/v NP‐40). Bound proteins were eluted by incubating the beads twice in 100 μL elution buffer (IPP150 with 4 mm biotin), digested with Trypsin gold (2 μg, 18 h) (Promega) and analysed by LC–MS/MS analysis (Heilmann *et al*., 2011). Protein identification was performed by using the MASCOT search engine using a *N. benthamiana* peptide database (NbDE) (Kourelis *et al*., 2019), supplemented with the peptide sequences of the tagged effectors.

### Yeast two‐hybrid assays

The yeast strain PJ69‐4a (James *et al*., 1996) was grown on non‐selective rich YPDA medium or on minimal medium (MM) supplemented with 20 mg/L L‐histidine HCL monohydrate (H), 100 mg/L L‐leucine (L), 20 mg/L L‐tryptophan (W), 20 mg/L adenine hemi sulphate (A), 30 mg/L lysine‐HCl (K), 20 mg/L methionine (M), and 20 mg/L uracil (U) (Gietz and Woods, 2002). For the selection of auxotrophic growth, one or more amino acids were omitted from the MM medium. Yeast transformation was performed by lithium acetate and polyethylene glycol 3350 methods (Gietz and Woods, 2002). To select for presence of bait and prey plasmids, transformed cells were grown for 4 to 5 days on MM–WL at 30 °C. 5 μL of resuspended cells were spotted on MM–WL, MM–WLH and MM–WLA plates to assess protein–protein interactions. Plates were imaged after 3 days of incubation at 30 °C.

Protein accumulation of TPLs and SIX8 fused to ‐AD or ‐BD was assessed by Western blot analysis. Proteins were extracted using the Urea/SDS method from the Clontech Yeast Protocols Handbook (https://www.takarabio.com). To detect fusions proteins, anti‐AD and anti‐BD monoclonal antibodies (Clontech 630402, Clontech 630403) were used, at a 1 : 2000 and 1 : 2500 dilution, respectively. In both cases, a secondary goat‐anti‐mouse antibody (Pierce 31430) was used in a 1 : 5000 dilution. Upon addition of commercial ECL solution (Amersham RPN22323), chemiluminescent signal was detected on light‐sensitive X‐ray film (Fuji RX) or on a Chemidoc MP imaging system.

### Bi‐molecular fluorescence complementation assay

*Agrobacterium tumefaciens* strain GV3101 was transformed with *pDEST‐VYNE(R)* ^*GW*^ *::TPL1* to ‐*TPL5*, *pDEST‐SCYCE(R)* ^*GW*^ *::SIX3* or *‐SIX8* and *pGWB454::NLS‐mCherry*. Equal amounts of *A. tumefaciens* transformed with (1) pDEST‐VYNE(R)^GW^ containing a specific *TOPLESS* homologue, (2) pDEST‐SCYCE(R)^GW^ containing either *SIX3* or *SIX8* and (3) pGWB454::*NLS‐mCherry* were mixed to an OD_600_ of 1.0 and infiltrated in the same leaf. Leaves were harvested at 24 hpi for fluorescence imaging of epidermal cells (LSM510, Zeiss, 40×/1.2 and 20×/0.75 objectives). The fluorophores were excited at 488 nm (GFP) or 543 nm (RFP) and emission was detected using a band‐pass filter (505–530 nm or 585–615 nm, respectively). The GFP intensity of the resulting images was analysed using an ImageJ script (Data S2). The data were log_10_ normalized before statistical analysis. Accumulation of the c‐myc‐tagged TPLs and HA‐tagged SIX8 and SIX3 effectors was assessed by immunoblotting. Proteins were extracted from four leaf discs (4 mm) as described (Cao *et al*., 2018). To detect the fusions proteins, anti c‐myc (α‐myc, 9E10, Roche 11667149001, 1 : 1000 dilution) and anti‐HA monoclonal antibodies (α‐HA 3F10, Roche 11867423001, 1 : 2000 dilution) were used. In both cases, a secondary goat‐anti‐mouse antibody (Pierce 31430) was used in a 1 : 5000 dilution. Chemiluminescence was detected using a Chemidoc MP imaging system.

### Gene editing of *TPL1* and *TPL2* in tomato

For CRISPR‐Cas9‐mediated gene editing a GoldenGate‐compatible cloning kit was used (Cermak *et al*., 2017). Four gRNAs were designed using CRISPR‐P, two gRNAs per *TPL* gene (Table S5). These gRNAs were assembled in a polycistronic *CmYLCV*_*Pro*_::(gRNA:Csy4)^4^ cassette using GoldenGate‐mediated directional cloning with *Sap*I in pDIRECT22C (Addgene 91135) according to protocol 3. The resulting vector pDIRECT22C_T4G was transformed into *A. tumefaciens* strain EHA105 and used for transformation of tomato cotyledons and regenerative *in vitro* culturing of explants on callus‐inducing media as described (Cortina and Culiañez‐Macia, 2004). Kanamycin‐resistant tomato plantlets were genotyped for the presence of the transgene and for *TPL1* and *TPL2* gene edits using primers FP8839/FP8840 and FP8841/FP8842, respectively (Table S4). T_1_ plants were screened for absence of the T‐DNA construct and for homozygous gene edits in ±300–400 bp fragments spanning the gRNA target sequences. The raw sequence reads (.abi files) were analysed using the ICE Synthego webtool to reveal edits (Hsiau *et al*., 2018). Out of 67 T_0_ plants analysed, eight plants (15%) were edited only in *tpl1*, 27 plants (51%) were edited only in *tpl2* and 5 plants were edited in both (7%). For this study, two knockout alleles for *TPL1* (a −10 bp [*tpl1‐1*] and −2 bp deletion [*tpl1‐2*]) were used, while for *TPL2* four independent alleles were selected (a −5 bp (*tpl2‐1*), −2 bp (*tpl2‐2*), −1 bp (*tpl2‐3*), −3 bp deletion (*tpl2‐4*) (Figure 2). Three different *tpl1;tpl2* double mutants were identified carrying small indels in both genes (*tpl1‐1;tpl2‐4, tpl1‐2;tpl2‐2* and *tpl1‐2;tpl2‐3*).

### Phenotypic analysis of *tpl* tomatoes

Four tomato plants of each *tpl* mutant line and cv. Motelle were grown in a greenhouse for 20 weeks (25/23 °C, 16/8 h day/night). Plants were photographed every week to monitor their development. Ripe tomatoes were harvested weekly, and fruit and seed yield were assessed by weighing.

### Tomato seedling grafting

Tomato seedlings were grown for 16 days in a greenhouse (25/23 °C, 16/8 h day/night) after which they were grafted using Grafting Cassettes™ (GRA&GREEN Inc). In short, seedlings were cut below the cotyledons and the scions were placed on the rootstocks of two *tpl* double mutants (*tpl1‐2,tpl2‐2* or *tpl1‐2,tpl2‐3*) (Figure 1). To facilitate recovery, the grafted seedlings were placed inside a plastic propagator to attain high humidity (>90%) for 12 days. During the first week, the propagators were covered with paper to reduce evaporation, after 9 days the vents were opened to equilibrate the humidity to ambient levels (50%–70% RH).

### Tomato and Arabidopsis disease assays

Ten‐day‐old greenhouse‐grown tomato seedlings (25 °C, 16/8 h light/dark cycle) were uprooted and inoculated with Fusarium by the root‐dip method by placing them for 5 min in a Fol029 spore suspension (Rep *et al*., 2005). The concentrations used were either 1 × 10^7^ spores/mL or 1 × 10^6^ spores/mL for grafted plants, as described (Gawehns *et al*., 2014; Rep *et al*., 2004). 3 weeks post‐inoculation, disease symptoms were scored (Rep *et al*., 2004). *V. dahliae* disease assays were performed on ten‐day‐old tomato seedlings using the root‐dip inoculation method as described previously (Fradin *et al*., 2009). At 14dpi, all plants were photographed from the top, resulting images were analysed in ImageJ to measure the canopy area. *P. syringae* assays were performed with the DC3000 isolate as described (Hassan *et al*., 2020). In short, tomato plants were germinated on ½ MS agar plates and grown for 10 days. Thereafter, seedlings were submerged in a bacterial inoculum (OD600 0.005). 4 days after inoculation, single cotyledons were sampled, and surface sterilized for a bacterial count assay.

For the Arabidopsis Fusarium assays seedlings were grown in climate‐controlled chambers (20 °C, 11/13 h light/dark cycle, 70% relative humidity). 14‐day‐old seedlings were uprooted and placed for 5 min in a suspension of 1 × 10^6^ Fo5176 spores/mL (Thatcher *et al*., 2009). Thereafter, seedlings were re‐potted and cultivated in a climate chamber at 28 °C to facilitate infection (11/13 h light/dark cycle, 80% relative humidity). Disease symptoms were scored 2 weeks after inoculation (Di *et al*., 2017a).

## Funding

TA was supported by NWO (GSGT.2018.0001 to TA) and Dekker Chrysanten B.V., Deliflor Chrysanten B.V., Dümmen Orange B.V., ENZA zaden Benelux B.V., Rijk Zwaan Nederland B.V. and Royal van Zanten B.V. FG and FT were supported by the Centre for BioSystems Genomics, a Netherlands Genomics Initiative and FT obtained support from the NWO‐Earth and Life Sciences funded VICI Project No. 865.14.003. MR and MdS were supported by the Dutch Technology Foundation (STW). The University of Amsterdam supported HvdB and HD. Manipulation of susceptibility to achieve disease resistance (NWO‐STW).

## Conflicts of interest

The authors declare that they have no conflicts of interest.

## Author contributions

TA generated the gene‐edited tomato lines and performed the grafting and all Fo, Pst and Vd disease assays. FG executed the pull‐down assay. MdS did BiFC and yeast two‐hybrid analysis. TA, FT and HvdB wrote the manuscript with input from the other co‐authors. HvdB, MR and FT supervised the research project.

## Supporting information


**Figure S1** Expression of *TPL1* and *TPL2* is relatively high in tomato root tissues.
**Figure S2** Accumulation of the proteins TPL1 to TPL5, SIX8 and SIX3 in the BiFC analyses following transient expression in *Nicotiana benthamiana* leaves.
**Figure S3** Accumulation of the tomato proteins TPL1 to TPL5 in the yeast two‐hybrid assays.
**Figure S4** Tomato *tpl1;tpl2* mutants have larger flowers.
**Figure S5** Tomato fruits of tomato tpl mutants.
**Figure S6** Fruit and seed yield of tomato *tpl1, tpl2* and *tpl1;tpl2* mutants.
**Figure S7** Susceptibility to *Verticillium dahliae* isolate Dvd‐S26 is unaffected in *tpl1;tpl2* mutants.
**Figure S8** Susceptibility to *Pseudomonas syringae* pv. tomato DC3000 is not altered in tomato *tpl1;tpl2* mutants.
**Figure S9** Susceptible tomato scions grafted on *tpl1;tpl2* rootstocks show a reduction of vasculature colonization by Fol029.
**Figure S10** Accumulation of Arabidopsis TPL and TPR1 to TPR4 in yeast two‐hybrid assays.
**Table S1** *F. oxysporum* f. sp. containing *SIX8* homologue(s) and their corresponding hosts.
**Table S2** Genotypes of tomato *tpl* mutants used in this study.
**Table S3** Plasmids used in this study.
**Table S4** Primers used in this study.
**Table S5** Guide RNAs used for gene‐editing tomato *TPL1* and *TPL2*.
**Data S1** Peptide hits of the SIX8‐pulldown in the NbDE proteome database.
**Data S2** ImageJ script for BiFC analysis.
