# Supplementary material for: Specific members of the TOPLESS family are susceptibility genes for Fusarium wilt in tomato and Arabidopsis
Source: Plant Biotechnol J. 2023 Oct 11;22(1):248–61. doi: 10.1111/pbi.14183 (PMC10754003; doi:10.1111/pbi.14183)
Supplement: Supplementary file 1 — Figure S1 Expression of TPL1 and TPL2 is relatively high in tomato root tissues. Figure S2 Accumulation of the proteins TPL1 to TPL5, SIX8 and SIX3 in the BiFC analyses following transient expression in Nicotiana benthamiana leaves. Figure S3 Accumulation of the tomato proteins TPL1 to TPL5 in the yeast two‐hybrid assays. Figure S4 Tomato tpl1;tpl2 mutants have larger flowers. Figure S5 Tomato fruits of tomato tpl mutants. Figure S6 Fruit and seed yield of tomato tpl1, tpl2 and tpl1;tpl2 mutants. Figure S7 Susceptibility to Verticillium dahliae isolate Dvd‐S26 is unaffected in tpl1;tpl2 mutants. Figure S8 Susceptibility to Pseudomonas syringae pv. tomato DC3000 is not altered in tomato tpl1;tpl2 mutants. Figure S9 Susceptible tomato scions grafted on tpl1;tpl2 rootstocks show a reduction of vasculature colonization by Fol029. Figure S10 Accumulation of Arabidopsis TPL and TPR1 to TPR4 in yeast two‐hybrid assays. Table S1 F. oxysporum f. sp. containing SIX8 homologue(s) and their corresponding hosts. Table S2 Genotypes of tomato tpl mutants used in this study. Table S3 Plasmids used in this study. Table S4 Primers used in this study. Table S5 Guide RNAs used for gene‐editing tomato TPL1 and TPL2. Data S1 Peptide hits of the SIX8‐pulldown in the NbDE proteome database. Data S2 ImageJ script for BiFC analysis. [file PBI-22-248-s001.zip › S_Data2_R1.docx]

ROOT_DIRECTORY = “/path/to/images/directory”;

files = getFileList(ROOT_DIRECTORY);

for (i = 0; i < files.length; i++) {

file = files[i];

if(endsWith(file, ".lsm")) {

calculateIntensity(ROOT_DIRECTORY, file, "C2");

}

}

function calculateIntensity(directory, filename, channel) {

open(directory + filename);

run("Split Channels");

selectWindow("C2-" + filename);

run("Duplicate...", "title=Duplicate-C2-" + filename);

run("Median...", "radius=4");

setAutoThreshold("Default dark");

run("Threshold...");

setThreshold(50, 255);

setOption("BlackBackground", false);

run("Convert to Mask");

run("Analyze Particles...", "size=40-200 circularity=0.90-1.00 show=Outlines add");

roiManager("Show All with labels");

roiManager("Show All");

selectWindow("Drawing of Duplicate-C2-" + filename);

run("Save", "save=[" + directory + replace(filename, ".lsm", "_mask.tif") + "]");

close();

run("Set Measurements...", " mean display redirect=None decimal=3");

selectWindow(channel + "-" + filename);

n = roiManager("count");

for(i = 0; i < n; i++) {

roiManager("select", i);

roiManager("Measure");

}

run("Save", "save=[" + directory + replace(filename, ".lsm", "_" + channel + ".tif") + "]");

roiManager("reset");

}
